# Development of Controllable Perfusion Culture Scaffolds Using Multi-Channel Collagen Gels: Effects of Gelation Conditions on Channel Formation and Media Supply

**DOI:** 10.3390/polym17030287

**Published:** 2025-01-23

**Authors:** Mareni Arishima, Ryota Haraguchi, Hidetaka Kawakita, Shigehisa Aoki, Yushi Oishi, Takayuki Narita

**Affiliations:** 1 Department of Chemistry and Applied Chemistry, Saga University, Saga 840-8502, Japan; 2 Department of Pathology and Microbiology, Saga University, Saga 840-8501, Japan

**Keywords:** tissue engineering, collagen scaffold, perfusion culture, multi-channel structure, cell proliferation, gelation conditions

## Abstract

The development of scaffold materials that effectively mimic the extracellular matrix while enabling controlled nutrient delivery remains a critical challenge in tissue engineering. Multi-channel collagen gels (MCCGs), which form through the competition between gelation and phase separation, have emerged as promising scaffolds due to their self-organized vessel-like structures. However, a systematic understanding of the relationship between the gelation conditions and functional properties is limited. In this study, MCCGs were developed as controllable perfusion culture scaffolds by investigating the effects of carbonate buffer concentration on channel formation, permeation behavior, and cell proliferation. MCCGs were prepared using different carbonate buffer concentrations (12.5, 25, and 50 mM), with 25 mM producing optimal channel formation, characterized by an approximately 60% channel area fraction and uniform distribution. Permeation studies revealed that fluid transport through MCCGs is governed by a complex interplay between capillary phenomena and hydraulic pressure, whose relative dominance shifts with flow rate: capillary action dominates at low flow rates (2.5 mL/h), whereas hydraulic pressure becomes the primary driver at higher rates (5.0–10.0 mL/h). Cell proliferation assessments demonstrated that MCCGs prepared with 25 mM carbonate buffer provided the most favorable microenvironment, achieving superior cell growth over 168 h through balanced media supply and cell adhesion area. This optimization approach through buffer concentration adjustment offers a cost-effective and scalable method for developing perfusion culture scaffolds, advancing both the fundamental understanding of functional gel systems and practical applications in tissue engineering and regenerative medicine.

## 1. Introduction

Research on scaffold materials in tissue engineering has been developing rapidly, driven by the increasing demand for advanced biomaterials that support tissue regeneration and growth [1,2]. The global scaffold technology market was worth USD 1.1 billion in 2020 and is expected to continue growing [3]. In tissue engineering and regenerative medicine, scaffolds are essential components that mimic the extracellular matrix (ECM) [4,5,6,7], providing crucial support for cell proliferation, migration, and differentiation [8,9,10]. Collagen, the most abundant structural protein in the human body, is a promising scaffold material. In particular, native bovine type I collagen has been widely used in tissue engineering applications due to its structural similarity to human collagen, excellent biocompatibility, and consistent quality. Recent studies have shown remarkable success with collagen-based, aligned, dense and porous gels for neural tissue regeneration [11,12,13] and cartilage repair. Additionally, drug delivery systems using collagen scaffolds have demonstrated sustained drug release capabilities [14,15] and improved therapeutic efficiency [16,17]. These diverse applications highlight the versatility of collagen as a biomaterial. Collagen is advantageous as a scaffold material because of its excellent biocompatibility, minimal immunogenicity, and ability to promote cell adhesion and proliferation [18,19]. Its adaptability enables mixing with other biomaterials and customization for specific applications [20,21,22,23]. In particular, collagen scaffolds with porous structures (multi-channel structures), such as sponge shapes and nanofiber mats, facilitate the exchange of nutrients and gases essential for cell survival and 3D cell culture [24,25,26,27,28]. These multi-channel structures directly influence scaffold functionality through their effects on permeability, medium supply efficiency, and cell proliferation [29,30,31].

Perfusion culture technology has become increasingly important for enhancing 3D culture systems by better replicating the in vivo environment [32,33,34]. This approach has demonstrated significant advantages over static culture, including doubled functional maintenance periods for primary hepatocytes [35,36] and improved cardiomyocyte function, which better mimics cardiac tissue [37,38]. A continuous supply of oxygen and nutrients promotes metabolic activity, whereas efficient waste removal contributes to maintaining cell health [38,39]. However, challenges remain in the development of scaffolds with controlled multi-channel properties and optimized perfusion characteristics. Current manufacturing techniques, such as 3D printing, electrospinning, and freeze-drying, have limitations in terms of cell penetration [40], low load-bearing capacity [41], manufacturing scaffolds for the regeneration of larger tissue volumes [42], and mechanical incompatibility with natural tissue [43].

Recently, multi-channel collagen gels (MCCGs) have emerged as a promising alternative scaffold system. MCCGs form through competition between gelation and phase separation [44,45], resulting in distinct and uniform vessel-like multi-channel structures within the collagen matrix [46,47]. This approach offers several advantages over conventional technologies, such as higher cost efficiency than 3D printing, reduced material waste, and simplified manufacturing without complex equipment. However, MCCGs also have certain limitations compared with 3D printing. While 3D printing enables precise control over scaffold architecture and the creation of complex geometries with predefined patterns, our MCCG approach relies on self-organization processes that can introduce some variability in channel formation. Additionally, 3D printing allows for the fabrication of scaffolds with specific external shapes for customized tissue engineering applications, whereas MCCGs are currently limited to simpler geometries. Furthermore, 3D printing facilitates the creation of hierarchical structures with multiple materials, which is challenging to achieve with our current MCCG method. Natural structure formation through self-organization also eliminates the need for multistep processing. Channel size and distribution can be controlled by adjusting gelation conditions [48,49], enabling optimization of nutrient supply in tissue engineering and drug release profiles.

This study focused on developing MCCGs as controllable perfusion culture scaffolds, addressing the challenges of multi-channel control in current scaffold technologies by evaluating the effects of gelation conditions on channel formation and media supply efficiency. Our approach is innovative in enabling precise control of multi-channel structures, which is difficult with conventional technologies, while simplifying the manufacturing process. The manufacturing method achieved by combining self-organization and phase separation enables efficient material use at lower costs than 3D printing and electrospinning technologies. Furthermore, by optimizing the carbonate buffer concentration, we constructed a 3D cell culture system that enables an efficient nutrient supply and cell proliferation by optimizing the MCCG structural characteristics. This approach provides new scaffold designs that can effectively promote cell proliferation, nutrient diffusion, and tissue formation.

Specifically, we investigated the following: 1. We prototyped a perfusion system and verified the feasibility of combining ease of manufacturing with advanced structural adjustment. 2. We measured the channel area and channel density of MCCGs prepared with different concentrations of the gelling agent to evaluate the possibility of multi-channel control in scaffold technology. 3. We clarified the differences in permeation rates in perfusion systems using MCCG scaffolds prepared with different concentrations of gelling agents and evaluated their effects on media supply efficiency. We also considered the reasons for these permeation rate differences based on the capillary phenomena and hydraulic pressure. 4. We elucidated the differences in proliferation rates of cells cultured on MCCG scaffolds prepared with different concentrations of the gelling agent and examined the effects of the MCCG microchannel structure on cell proliferation.

The findings of this study deepen the understanding of functional gel systems and expand the versatility of biopolymers for various applications, including tissue engineering, drug delivery, and cultured meat production. This demonstrates the adjustability of biopolymer-based scaffolds and their potential applications in medical and industrial fields.

## 2. Materials and Methods

### 2.1. Materials

Native Type I Collagen Acidic Solution (IAC-50, 5 mg/mL) was obtained from Koken Co., Ltd. (Tokyo, Japan). The collagen was dissolved in 1 mM HCl solution (pH 2.99). The collagen solution was used as received without further purification. The purity was greater than 95% total collagen, as determined by SDS-PAGE analysis, according to the manufacturer’s specifications. Riboflavin was purchased from FUJIFILM Wako Pure Chemical Corporation (Osaka, Japan). Carbonate buffer was prepared by dissolving carbonate powder from DKK-TOA Corporation (Tokyo, Japan) in 500 mL of distilled water.

### 2.2. Preparation of Multi-Channel Collagen Gels (MCCGs)

**Figure 1:**
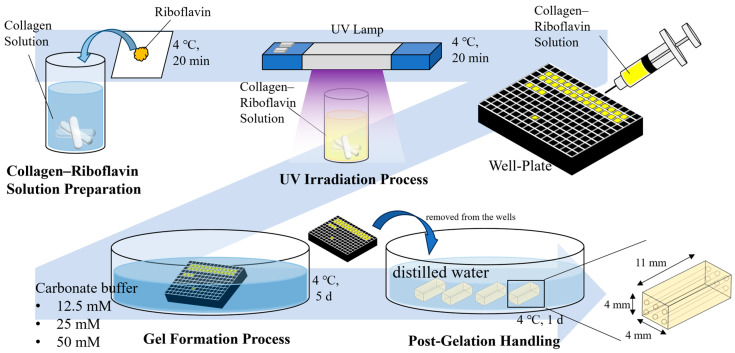
Preparation of multi-channel collagen gels (MCCGs).

Figure 1 illustrates the preparation process of MCCGs. Riboflavin (4.3 µmol) was added to 10 mL of Native Collagen Acidic Solution and was completely dissolved using a magnetic stirrer in a refrigerator maintained at 4 °C overnight. The resulting collagen–riboflavin solution was transferred to a vial and irradiated with ultraviolet light at 366 nm using a UV lamp 4 (8 W, Camag, Muttenz, Switzerland) at room temperature for 20 min. To ensure uniform UV irradiation of the collagen–riboflavin solution in the vial, the vial was rotated every 5 min during irradiation. The light source was positioned 15 cm above the room-temperature beaker, with a UV radiation intensity of 4.12 W/m^2^. The purpose of UV irradiation is to increase the viscosity of the pre-gel solution without achieving complete crosslinking, thereby controlling the gelation rate. After UV irradiation, the pre-gel solution was transferred to a syringe and 110 µL was injected into each well of a well plate (AS ONE Corporation, Osaka, Japan). The well plate was then immersed in carbonate buffer (12.5, 25, or 50 mM) and maintained at 4 °C for 5 days to form rectangular hydrogels measuring 4 mm × 4 mm × 11 mm in the well plate. A 5-day period was set to allow complete gelation and ensure sufficient time for hydrogel structure formation and stabilization. The formed gels were carefully removed using a needle and stored overnight in distilled water at 4 °C.

### 2.3. Characterization of Channel Structure

The multi-channel collagen gels were immersed in 5 × 10⁻^3^ M FITC solution (R&D Systems, Inc., Minneapolis, MN, USA) for 15 min and washed five times with distilled water to remove excess dye. The internal structures of the porous collagen gels were observed using an inverted fluorescence microscope (THUNDER; Leica Microsystems GmbH, Wetzlar, Germany). FITC-stained collagen gels were frozen at −80 °C, sliced with a razor parallel to the carbonate buffer diffusion surface, and their cross-sections observed. The fluorescence excitation and emission wavelengths were set at 480 and 520 nm, respectively. Fluorescence images were acquired using microscope-mounted analysis software(LAS X, ver.3.9.1.28433), and the channel area and density were quantified using ImageJ(ver.1.54h), an open-source image analysis software (National Institutes of Health, Bethesda, MD, USA). At least five images were analyzed for each sample to adequately capture the sample variation. The results were calculated as the mean channel diameter and the channel area fraction. The channel area fraction was determined by dividing the total channel area by the cross-sectional area of the gel. The standard deviation indicates the variation between the samples.

### 2.4. Fabrication of Perfusion Device

**Figure 2:**
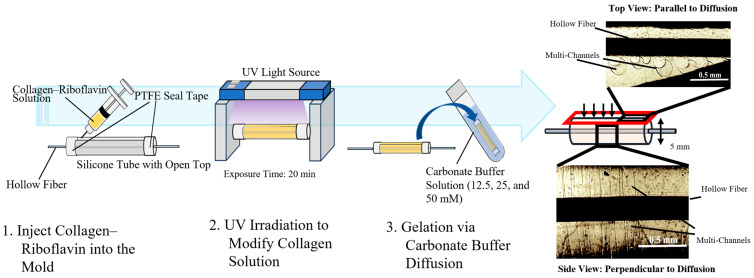
The process of preparation of perfusion device covered with multi-channel collagen gel (MCCG).

Figure 2 shows the method for fabricating a perfusion device using the MCCG as a cell scaffold for irrigation culture. A silicone mold (Monotaro; inner diameter, 12 mm; outer diameter, 16 mm; length, 25.5 mm) with an opening at the top was sealed at both ends using sealing tape. The collagen–riboflavin solution prepared for MCCG preparation was injected through the mold opening using a syringe (6 mL) with 330 µL per mold. A hollow fiber membrane (Kitz Microfilter Co., Ltd., Nagano, Japan) was positioned along the central axis of the mold, and UV irradiation was performed according to the aforementioned MCCG preparation protocol. The mold was then immersed in 400 mL of carbonate buffer (12.5, 25, or 50 mM) and held at 4 °C for seven days to form an MCCG scaffold with a hollow fiber membrane embedded in a cylindrical collagen matrix. The pre-gel collagen–riboflavin solution underwent UV-initiated crosslinking in the mold, increasing the solution viscosity. This enabled stable MCCG formation, even with a dilute carbonate buffer. The resulting hydrogel showed a structure with channels arranged radially (perpendicular to the fiber axis), as shown in Figure 2, facilitating the radial transport of the culture medium from the hollow fiber lumen through the channels to the gel surface. The formed MCCG scaffold was easily removed from the silicone mold and stored in distilled water at 4 °C for incorporation into the perfusion system.

### 2.5. Perfusion System Model Setup and Permeation Behavior Measurement

**Figure 3:**
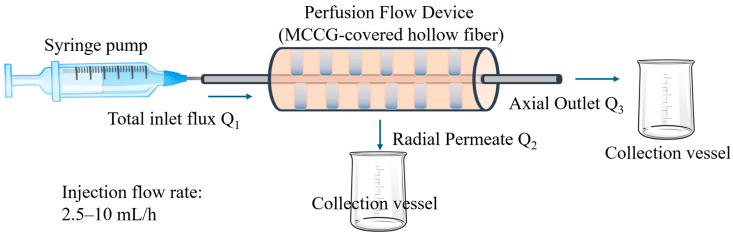
Schematic representation of the perfusion system using MCCG-covered hollow fiber.

A mass transport system was constructed to evaluate the permeation efficiency through MCCG. As shown in Figure 3, the experimental apparatus consisted of a syringe pump for fluid injection, perfusion device with a hollow fiber membrane embedded in collagen gel, a collection vessel for radial permeation flux, and a collection vessel for axial outlet flux from the fiber end. Distilled water was used as the process fluid for the baseline characterization of the MCCG-covered device. The perfusion flow rates (injection rates) through the hollow fiber were set at 2.5, 5.0, and 10 mL/h, with operations conducted at room temperature for 30 min.

This system mimics biological vascular networks by utilizing the hollow fiber as a main blood vessel and multi-channel structure within collagen gels as capillaries. In biological systems, cells receive nutrients and oxygen through mass transport through the interstitial space and capillary networks. Therefore, the rates of nutrient and oxygen transport are proportional to the volumetric flow in these regions. In this study, we quantified the radial permeation flux through the hollow fiber wall, corresponding to the mass transport to the interstitial and capillary regions. The radial permeation volume Q_2_ (volume of fluid permeating radially through the hollow fiber wall, with or without the MCCG coating) was determined by measuring the axial outflow volume Q_3_ using an analytical balance at predetermined time intervals and subtracting this value from the total inflow volume Q_1_. Thus, we quantified the permeation rate of distilled water through MCCG scaffolds prepared with different gelling agent concentrations to predict the efficiency of media supply to cell scaffolds. The permeate volume was calculated from the weight, assuming a density of 1 g/cm^3^. During the measurements, hollow fiber regions not covered by MCCG were sealed with waterproof tape to ensure permeation only through the MCCG-covered portion of the device. The obtained data were compared with the channel size and density characteristics from the channel structure evaluation and results from control samples of uncovered hollow fibers to assess the utility of MCCG structures as perfusion culture cell scaffolds.

### 2.6. Cell Proliferation Assessment

NIH/3T3 fibroblasts (JCRB IFO50019, obtained from the Japanese Collection of Research Bioresources Cell Bank, Ibaraki, Japan) were used to evaluate cell proliferation rates on MCCGs. This cell line was originally established from mouse embryonic fibroblasts by A. Hakura in 1985 and maintained in Eagle’s minimal essential medium supplemented with 10% fetal bovine serum. Prior to cell seeding, the MCCGs were sterilized by immersion in an 80% ethanol solution and subsequently washed three times with PBS. The washed MCCGs were placed at the bottom of cell culture inserts (Millicell-CM, Millipore, PICM01250, Bedford, MA, USA), and 500 µL of cell suspension (1 × 10^3^ cells/mL) prepared in medium supplemented with 10% bovine serum was seeded onto the MCCGs. To promote cell adhesion, the seeded MCCGs were incubated at 37 °C under 5% CO_2_, 20% O_2_ conditions for 5 h. After adhesion, the MCCGs were transferred to 30 mm plastic Petri dishes, 2 mL of 10% bovine serum medium was added, and the culture was continued for 6 days. The medium was changed daily and the culture was conducted under static conditions.

Cell proliferation was evaluated using a colorimetric CCK-8 assay kit, according to the manufacturer’s protocol. At specific time points (5 h after seeding, and 1 and 5 days after culture), 100 µL of medium and 10 µL of assay reagent were added to 96-well plates containing MCCGs. After 150 min of incubation, the absorbance was measured at 450 nm using a microplate reader (Luminoskan Ascent 5300170, Thermo Scientific, Waltham, MA, USA). Measurements beginning 5 h after seeding were used to calculate cell proliferation rates, with initial cell adhesion time as the baseline. Each experiment was repeated five times. Statistical analysis was performed using one-way analysis of variance (ANOVA), followed by Tukey’s post hoc test. Statistical significance was set at *p* < 0.05. Homogeneity of variance was confirmed by the F-test prior to conducting ANOVA.

## 3. Results and Discussion

### 3.1. Channel Structure of Multi-Channel Collagen Gels

**Figure 4:**
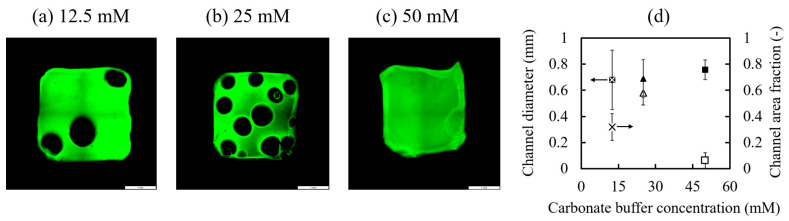
Microscope images showing typical short-axis cross-sections of MCCGs prepared with different concentrations of carbonate buffer: (**a**) 12.5 mM, (**b**) 25 mM, and (**c**) 50 mM. Scale bar: 1 mm. (**d**) Dependence of the channel diameter (closed symbols) and channel area fraction (open symbols) on the carbonate buffer concentration. The symbols ×, Δ, and □ represent 12.5, 25, and 50 mM samples, respectively. The arrows in the graph indicate the corresponding axis. Error bars indicate standard deviation (*n* = 6).

Figure 4a–c show representative fluorescence microscope images of the short-axis cross-section (perpendicular to the buffer diffusion direction) of the MCCGs prepared using different concentrations of carbonate buffer (12.5, 25, and 50 mM). These images visually demonstrate the impact of buffer concentration on the channel structure, particularly highlighting the differences in channel size, density, and distribution. These differences indicate that proper channel formation directly enhances scaffold functionality by improving the nutrient supply efficiency and promoting cell proliferation.

The observed MCCG structures exhibited different characteristics depending on buffer concentration. The average channel diameter was relatively unaffected by the buffer concentration, remaining at approximately 0.7 mm. However, the number of channels per unit area varied significantly with the buffer concentration. Gels prepared with 12.5 mM carbonate buffer showed approximately three channels, and those prepared with 25 mM showed approximately eleven channels, while gels prepared with 50 mM showed almost no channel formation. The channels were distributed nearly uniformly across the cross-section of the gel.

As shown in Figure 4d, the channel area fraction varied significantly with the buffer concentration. Gels prepared with 25 mM carbonate buffer showed more than twice the channel area fraction compared with those prepared with 12.5 mM, with channels occupying approximately 60% of the area. In contrast, MCCGs prepared with 50 mM carbonate buffer showed almost no channels, with most samples exhibiting a structure that was completely covered by gel.

MCCG structure is known to form through the competition between phase separation of the collagen solution and collagen gelation [45,47,49], with channels generated and growing during this process. At high carbonate buffer concentrations, the structure is fixed before the channels can macroscopically develop during the initial phase separation stage, resulting in minimal channel formation. The high number of channels in the 25 mM samples is attributed to the carbonate buffer acting as a quenching agent, destabilizing the system, and promoting channel nucleus formation. The 12.5 mM samples showed fewer channels compared with the 25 mM samples due to weaker quenching effects, making growth more dominant than nucleation. The buffer concentration dependence of the channel area fraction shown in this study aligns with previous reports [49], supporting the fact that MCCG structures were formed equivalently to known methods.

A high channel area fraction is expected to promote media flow in perfusion systems. Conversely, a decreased channel number density may limit the available surface area for cell adhesion, potentially affecting both cell proliferation and nutrient supply efficiency. These results demonstrate that the carbonate buffer concentration plays a crucial role in determining the internal structure of multi-channel collagen gels.

### 3.2. Permeation Behavior from Perfusion System Model

**Figure 5:**
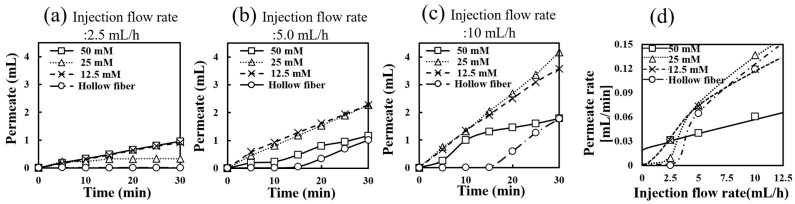
Flow rate-dependent permeate behavior of MCCG devices formed with different carbonate buffer concentrations. (**a**–**c**) Time course of cumulative permeate volume at different injection rates ((**a**): 2.5 mL/h, (**b**): 5.0 mL/h, (**c**): 10.0 mL/h). MCCG devices formed with 12.5 mM (×), 25 mM (∆), and 50 mM (☐) carbonate buffers were compared with hollow fiber controls without an MCCG covering (○). (**d**) Relationship between injection flow rate and permeate rate for each device, where permeate rates were calculated from the maximum slopes in (**a**–**c**).

Figure 5 shows the permeation amounts from MCCG devices formed with different carbonate buffer concentrations (12.5 mM, 25 mM, 50 mM) at different injection rates (2.5, 5.0, 10 mL/h), compared with hollow fiber controls (devices without MCCG coating).

In the low-injection-rate region of 2.5 mL/h (Figure 5a), permeation from the MCCG scaffolds remained relatively low regardless of the carbonate buffer concentration. Notably, while almost no permeate was observed from the hollow fiber in this injection rate region, the 50 mM condition with no channels showed slightly higher permeation amounts compared with 12.5 mM and 25 mM. These results indicated that the covered collagen gel promoted permeation in the low-injection-rate region.

In the medium-injection-rate region of 5.0 mL/h (Figure 5b), devices covered with MCCGs having multiple channels (formed with 12.5 mM and 25 mM carbonate buffers) showed high permeation rates. Conversely, devices formed with 50 mM carbonate buffer, which showed the fastest permeation rate in the low-injection-rate region, exhibited lower permeation rates than the other MCCG devices. In the case of hollow fibers without an MCCG coating (control group), almost no permeation occurred until 15 min; however, beyond 15 min, they showed permeation rates equivalent to those of devices covered with MCCGs and containing multiple channels. This trend indicates that the permeation flow through channels or hollow fiber pores becomes dominant in this injection rate region. Consequently, permeation rates from devices covered with gel containing no channels showed relatively lower rates.

At high injection flow rates of 10 mL/h (Figure 5c), the permeate volumes further increased, particularly in the 12.5 mM and 25 mM scaffolds. Gel scaffolds prepared with 25 mM carbonate buffer showed the highest permeate volume, followed by 12.5 mM. The 50 mM scaffold showed lower permeate volumes than the other devices. At high flow rates, the presence or absence of channels had a significant effect on the permeate volume. The permeation volume from the hollow fibers without the MCCG coating, similar to the 5.0 mL/h injection case, was hardly observed before 15 min of injection, but permeated at rates equivalent to MCCG-covered devices thereafter. The absence of permeation before 15 min can be explained by the surface energy required for the liquid to wet the polypropylene membrane wall surface, as the hollow fibers are made of hydrophobic polypropylene. This permeation limitation suggests that the capillary phenomena significantly influence the basic verification of the intended perfusion device. Meanwhile, covering the hollow fiber surface with a gel containing no channels appears to play a role in alleviating this resistance energy.

Graph 5d shows the permeation rates (maximum slopes from Figure 5a–c) as a function of injection rate (2.5, 5.0, and 10.0 mL/h) for hollow fibers covered with MCCGs prepared using different carbonate buffer concentrations and uncovered hollow fibers. All samples showed nonlinear increases in permeation rate with increasing flow rate, suggesting that the characteristics of each scaffold material influence fluid dynamics differently. Devices with channels prepared with 25 mM and 12.5 mM buffers showed the highest permeation rates, as mentioned above, demonstrating that channels effectively promote permeation volume corresponding to nutrient supply to cells.

The MCCGs prepared with 50 mM showed higher permeation rates than the other MCCG-covered devices at slow flow rates. However, this relationship is reversed at higher flow rates. Considering that 50 mM MCCGs have no channel formation and cellulose hollow fibers are hydrophobic, this permeation rate reversal can be explained by the nature of capillary phenomena and hydraulic pressure determining the permeation volume. Specifically, at slow flow rates, capillary phenomena are dominant, making permeation advantageous for devices without channels that are more heavily covered with collagen gel. Conversely, at fast flow rates, the hydraulic pressure becomes dominant, increasing the permeation volume from the MCCGs with channels and hollow fibers.

### 3.3. Theoretical Analysis of Permeation Rate Based on Capillary Phenomena and Dynamic Pressure

To determine the permeate rate through the membrane wall of a hollow fiber under perfusion conditions, the combined effects of capillary action and dynamic pressure were considered. The analysis incorporates the structural parameters of the hollow fiber and the physical properties of the fluid. We let r denote the inner diameter of the hollow fiber, *d* the diameter of the micropores in the membrane wall, *γ* the surface tension of the fluid, *θ* the contact angle between the fluid and the pore wall, *η* the dynamic viscosity of the fluid, *ρ* the density of the fluid, *X* the perfusion flow rate (injection flow rate) of the fluid within the hollow fiber, and *L* the thickness of the membrane wall.

The rate of fluid permeation due to capillary action is derived from the Lucas–Washburn equation, which is widely used to model capillary flow in porous media [50,51,52]. The capillary-driven rate is expressed as $v_{\mathrm{cap}}=\frac{1}{2}\sqrt{\frac{\gamma dcos\theta }{2\eta t}}$, where *t* represents the time elapsed since the onset of permeation. This rate is governed by the pore diameter *d*, contact angle *θ*, and the fluid properties such as surface tension *γ* and viscosity *η*.

In addition to capillary action, the permeate is influenced by dynamic pressure arising from the perfusion flow. The dynamic pressure inside the hollow fiber is given by $P_{\mathrm{dyn}}=\frac{1}{2}\rho X^{2}$, according to the Bernoulli principle [53,54]. This pressure drives fluid through the micropores, resulting in a rate that can be expressed using Poiseuille’s law as $v_{\mathrm{dyn}}=\frac{d^{2}P_{\mathrm{dyn}}}{32\eta L}$. Substituting, we obtain $v_{\mathrm{dyn}}=\frac{d^{2}\rho X^{2}}{64\eta L}$.

The total permeate rate is the sum of the capillary- and dynamic pressure-induced velocities. Combining these contributions, the total rate is expressed as $v_{\mathrm{total}}=v_{\mathrm{cap}}+v_{\mathrm{dyn}}$. Substituting the expressions for $v_{\mathrm{cap}}$and $v_{\mathrm{dyn}}$, the total rate becomes $v_{\mathrm{total}}=\frac{1}{2}\sqrt{\frac{\gamma dcos\theta }{2\eta t}}+\frac{d^{2}\rho X^{2}}{64\eta L}$.

To calculate the total permeate flow rate from the entire hollow fiber, the number of micropores *N* in the membrane wall was considered. Assuming uniform pore distribution, *N* is estimated as $N=\frac{\pi r^{2}}{\pi {\left(\frac{d}{2}\right)}^{2}}=\frac{4r^{2}}{d^{2}}$. The total permeate flow rate is then $V_{\mathrm{total}}=N\cdot v_{\mathrm{total}}$. Substituting $v_{\mathrm{total}}$ and *N*, the total flow rate is written as $V_{\mathrm{total}}=\frac{4r^{2}}{d^{2}}(\frac{1}{2}\sqrt{\frac{\gamma dcos\theta }{2\eta t}}+\frac{d^{2}\rho X^{2}}{64\eta L})$. Simplifying further, this becomes $V_{\mathrm{total}}=2r^{2}(\frac{1}{d^{2}}\sqrt{\frac{\gamma dcos\theta }{2\eta t}}+\frac{\rho X^{2}}{16\eta L})$.

**Figure 6:**
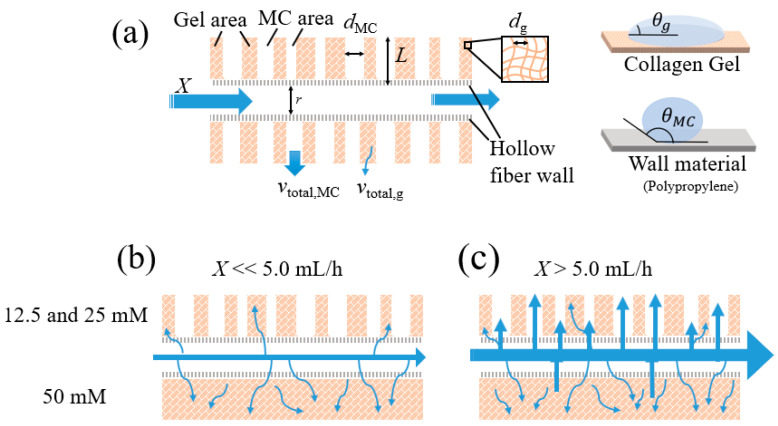
(**a**) Schematic representation of the MCCG-covered hollow fiber structure, highlighting the gel area (g) and microporous cellulose (MC) area, along with the associated structural parameters (micropore diameters dg, contact angles *θ*g and *θ*_MC_). The diagram illustrates the mechanisms of capillary action and dynamic pressure contributing to the total permeate flow velocity (*v*_total,g_ and *v*_total,MC_). (**b**) Fluid permeate behavior at low injection velocities (*X* ≪ 5.0 mL/h), where capillary-driven flow dominates, especially in gel regions with smaller pore diameters (*d*_g_) and lower contact angles (*θ*_g_). (**c**) Permeate behavior at high injection velocities (*X* > 5.0 mL/h), where dynamic pressure effects (*P*_dyn_ ∝ *X*^2^) become significant, leading to enhanced flow in MC regions with larger pore diameters (*d*_MC_) and higher contact angles (*θ*_MC_).

Our system consists of an MCCG layer covering the hollow fiber wall. As depicted in Figure 6a, the hollow fiber wall is divided into two distinct regions: the gel area (g) and MC area (MC). In these two regions, the micropore diameters (*d*_g_ and *d*_MC_), contact angles (*θ*_g_ and *θ*_MC_), and number of pores (*N*_g_ and *N*_MC_) differ significantly.

The total permeate flow rate (*V*_total_) through the MCCG-covered hollow fiber wall can be expressed as the sum of the contributions from both the gel and MC regions. For each region, the permeate flow rate is determined by the product of the number of micropores (*N*) and the total permeate velocity (*v*_total_), which incorporates both capillary-driven and dynamic pressure-induced components.

The total rate for the gel and MC regions is given by the following expressions:

$$v_{\mathrm{total},g}=\frac{1}{2}\sqrt{\frac{\gamma d_{g}cos\theta_{g}}{2\eta t}}+\frac{d_{g}^{2}\rho X^{2}}{64\eta L},\;v_{\mathrm{total},\mathrm{MC}}=\frac{1}{2}\sqrt{\frac{\gamma d_{\mathrm{MC}}cos\theta_{\mathrm{MC}}}{2\eta t}}+\frac{d_{\mathrm{MC}}^{2}\rho X^{2}}{64\eta L}.$$

The total permeate flow rate is then expressed as follows:

$$V_{\mathrm{total}}=N_{g}\cdot v_{\mathrm{total},g}+N_{\mathrm{MC}}\cdot v_{\mathrm{total},\mathrm{MC}}$$

This formulation accounts for the differences in pore diameter ($d_{g},d_{\mathrm{MC}}$), contact angle ($\theta_{g},\theta_{\mathrm{MC}}$), and number of pores ($N_{g},N_{\mathrm{MC}}$) between the two regions. It also incorporates the combined effects of capillary action and dynamic pressure on the permeate, thereby providing a foundation for analyzing and optimizing the fluid transport characteristics of the membrane wall.

Using the derived equation, the crossover points observed in the cumulative permeate flow curves in Figure 5 arise from the transition in dominance between capillary-driven and dynamic pressure-driven flows as the injection rate increases. At low injection velocities, capillary action is the primary driver, with the gel region contributing more significantly due to its smaller pore size ($d_{g}$) and lower contact angle ($\theta_{g}$), as shown in Figure 6b. The contact angle of polypropylene, the raw material for hollow fibers, is usually in the range of 90–98 degrees [55]. In this regime, systems dominated by gel pores exhibit higher permeate flow compared with MC-dominated systems, where the larger pore size ($d_{MC}$) and higher contact angle ($\theta_{MC}$) reduce the capillary effect. As the injection rate increases, dynamic pressure ($P_{\mathrm{dyn}}\propto X^{2}$) becomes the dominant factor. Since the dynamic pressure-driven flow scales with $d^{2}$, systems dominated by MC pores experience a rapid increase in permeate flow, eventually surpassing the gel-dominated systems (Figure 6c). The crossover points represent the transition between these regimes, as determined by the relative pore sizes, contact angles, and ratio of gel and MC pores. This interplay highlights how the injection rate modulates the balance between the capillary and dynamic pressure effects in systems with heterogeneous membrane structures.

Despite the simplicity of the derived permeation rate formula, it effectively explained the complex permeation behavior observed in our perfusion system model. Our findings underscore the importance of considering multiple factors in perfusion cultures using a 3D porous cell scaffold. Specifically, in addition to the medium injection rate and pore size, the interplay between the scaffold and medium affinity (e.g., contact angle) and the capillary phenomenon within the gel scaffold must be considered. Notably, the relationship between porosity and permeability in cell scaffolds remains ambiguous, with reports indicating that scaffolds with low porosity do not necessarily exhibit low permeability [56]. The perspective we present here offers a valuable clue for unraveling this complex relationship and advancing our understanding of scaffold permeability.

### 3.4. Cell Proliferation Rate

**Figure 7:**
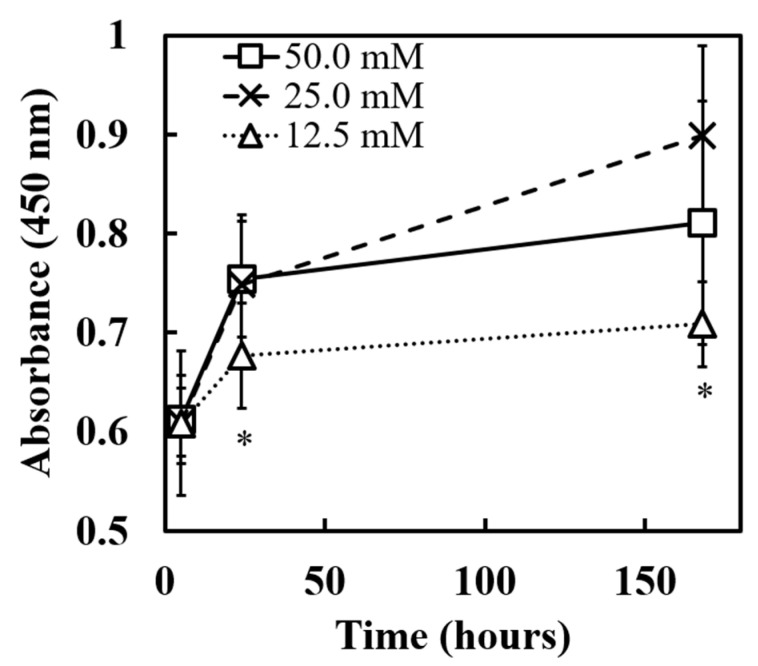
Cell proliferation was assessed using the CCK-8 assay. Values represent means from five independent experiments comparing MCCGs prepared with different carbonate buffer concentrations at 5, 24, and 168 h. Statistically significant differences between groups (*p* < 0.05) were determined by one-way ANOVA followed by Tukey’s post hoc test and are indicated by asterisks (*).

Figure 7 shows the results of cell proliferation evaluation on MCCGs prepared with different carbonate buffer concentrations (50.0 mM, 25.0 mM, 12.5 mM) using the CCK-8 assay. Up to 24 h from the start of the experiment, the absorbance (450 nm) increased rapidly (from approximately 0.6 to around 0.75) under all conditions, followed by a gradual increase up to 168 h. Particularly, the 25.0 mM condition reached the highest absorbance (approximately 0.9) after 168 h, demonstrating the most effective cell proliferation. The 50.0 mM condition showed moderate proliferation (absorbance about 0.8), while the 12.5 mM condition showed relatively lower proliferation rates (absorbance about 0.7).

These results are closely related to the microstructure of the MCCGs. The highest cell proliferation observed in the 25.0 mM condition can be attributed to its optimal structural characteristics, with uniformly distributed channels and a channel area fraction of approximately 60%. This structure is believed to achieve a balance between efficient media supply and a sufficient cell adhesion area.

Conversely, in the 12.5 mM condition, the lower number of channels and relatively smaller channel area fraction likely resulted in reduced media supply efficiency, consequently inhibiting cell proliferation. Additionally, under the 50.0 mM condition, the almost complete absence of channels resulted in a gel-dominant structure, limiting the media supply and leading to reduced cell proliferation compared with the 25.0 mM condition.

Focusing on temporal progression, the rapid proliferation observed across all conditions up to 24 h likely reflects the initial cell attachment and proliferation on available surfaces. The subsequent differences in proliferation rates up to 168 h are thought to reflect differences in nutrient supply efficiency owing to varying channel structures under each condition. The highest proliferation shown under the 25.0 mM condition particularly suggested that this condition provides an optimal microenvironment for long-term cell culture.

While our CCK-8 assay results demonstrate overall cell viability and proliferation, we acknowledge that this method alone cannot definitively determine the spatial distribution of cell growth within the MCCG structure. However, previous studies with single-channel collagen gels (Ishibashi et al., 2023) [48] have demonstrated successful cell proliferation within channel structures similar to those in our MCCGs.

Although the current study demonstrates the biocompatibility and overall cell proliferation support of MCCGs, further investigation using advanced imaging techniques such as fluorescence microscopy would be valuable to definitively establish the spatial distribution of cell growth within the channel structures. Such studies would provide important insights into how cells utilize the channel architecture and could help optimize the scaffold design for specific tissue engineering applications.

## 4. Conclusions

This study successfully developed and characterized MCCGs as controllable perfusion culture scaffolds, demonstrating their potential for tissue engineering applications. The key findings and conclusions are as follows:

First, we established that carbonate buffer concentration plays a crucial role in determining the internal structure of MCCGs. Particularly, gels prepared with 25 mM carbonate buffer exhibited optimal channel formation with approximately 60% channel area fraction, while maintaining uniform channel distribution. This finding provides a practical method for controlling scaffold architecture without complex equipment or processes.

Second, our perfusion system model revealed that the permeation behavior through MCCGs is governed by both capillary phenomena and hydraulic pressure, with their relative importance varying according to the flow rate. At low flow rates (2.5 mL/h), capillary action dominates the permeation process, while at higher flow rates (5.0–10.0 mL/h), hydraulic pressure becomes the primary driver of fluid transport. This understanding enables better control of nutrient delivery in tissue engineering applications.

Third, cell proliferation studies demonstrated that MCCGs prepared with 25 mM carbonate buffer showed superior cell growth over 168 h, achieving the highest proliferation rates among all tested conditions. However, additional microscopic characterization would be valuable to fully understand how cells distribute and grow within the scaffold’s channel structure. This optimal performance can be attributed to the balance between efficient media supply through the channel network and adequate surface area for cell adhesion.

These results establish MCCGs as promising platforms for tissue engineering applications, offering several advantages over conventional scaffold technologies.

- Controllable structure formation through simple adjustment of buffer concentration.
- Cost-effective manufacturing process utilizing self-organization principles.
- Efficient nutrient delivery system combining capillary and pressure-driven transport.
- Optimal support for cell proliferation and growth.

The insights gained from this study contribute to the fundamental understanding of functional gel systems and expand the potential applications of biopolymer-based scaffolds in the medical and industrial fields. Future research could focus on scaling up these systems for larger tissue constructs and investigating their performance with different cell types and tissue-specific applications.

## Data Availability

Data are contained within the article.
